# Severe Acute Global Systolic Dysfunction Secondary to Dengue Myocarditis: A Case Report

**DOI:** 10.7759/cureus.109806

**Published:** 2026-05-28

**Authors:** Rafi Ullah, Laiba Ashfaq, Yasir Iqbal

**Affiliations:** 1 Internal Medicine, West Cumberland Hospital, Whitehaven, GBR; 2 Internal Medicine, Pakistan Atomic Energy Commission (PAEC) General Hospital, Islamabad, PAK; 3 Acute and General Medicine, Queen Elizabeth Hospital Birmingham, Birmingham, GBR

**Keywords:** dengue fever/complications, dengue myocarditis, fluid overload, heart failure, hypotension treatment

## Abstract

We describe the case of a 47-year-old woman with laboratory-confirmed dengue fever who developed acute myocarditis during the critical phase of illness. On presentation, along with her initial symptoms of fever, headache, and myalgia, she also reported central chest discomfort and exertional dyspnoea. A standard transthoracic echocardiogram demonstrated severe left ventricular systolic dysfunction with an ejection fraction of 20%. She was treated in the intensive care unit with cautious fluid because of the risk of volume overload in dengue with cardiac involvement. She was able to come off inotropes after the acute phase of illness, and echocardiography at the three-month follow-up revealed improved ejection fraction to 40% and resolution of symptoms. This case points out the need for early detection of myocardial involvement in dengue infection and the importance of judicious fluid management to avert cardiac decompensation and improve prognosis.

## Introduction

Dengue fever is an arthropod-borne viral infection transmitted primarily by Aedes aegypti and other Aedes species. It is a highly prevalent infectious disease in Southeast Asia [1]. WHO has categorized dengue infection into two broad categories, namely dengue with or without warning signs and severe dengue [2]. Dengue fever is known to cause cardiac complications ranging from pericardial effusions, myocarditis, atrioventricular block, ectopic ventricular beats, and atrial fibrillation [3].

Myocardial involvement in dengue fever can cause refractory shock (usually a combination of hypovolemic and cardiogenic), and that is one of the difficult situations to be faced with as a clinician [4]. Dengue fever is usually a fluid deficit state (either due to simple dehydration or third-space fluid loss), and fluid replacement is the mainstay of treatment [5]. On the other hand, cardiac involvement in dengue infection puts patients at risk of fluid overload, thus posing diagnostic and therapeutic challenges. Therefore, it is paramount that cardiac involvement is recognized early and managed accordingly. Our case report is to emphasize the importance of vigilance regarding cardiac involvement in dengue fever.

## Case presentation

A 47-year-old South Asian woman with no significant past medical history presented with a five-day history of high-grade fever associated with myalgia and headache, followed by a two-day history of cough, exertional shortness of breath, and central chest discomfort. There was no history of travel, mucosal bleeding, haematuria, epistaxis, or bleeding from any site.

On admission, her blood pressure was 118/72 mmHg without postural drop, heart rate 110 beats/minute, temperature 100°F, and oxygen saturation 87% on room air. Capillary blood glucose was 120 mg/dL. She appeared dehydrated and had petechial rashes over the extensor surfaces of both arms and legs. There were bilateral basal crepitations on lung auscultation, with normal heart sounds, an elevated jugular venous pressure, and pedal oedema. There were no signs of meningeal irritation or focal neurological deficit.

Investigations

Initial laboratory investigations revealed a platelet count of 182×10⁹/L, leukopenia (total leucocyte count 1.9×10⁹/L), and haemoglobin 10.7 g/dL (haematocrit 32%). Dengue NS1 antigen was positive, while the malaria parasite smear was negative. C-reactive protein was 1.3 mg/dL, and procalcitonin was within normal limits. Liver and renal function tests and serum electrolytes were normal. Serial complete blood counts showed a progressive fall in platelet count to 75×10⁹/L by day 7 of illness, with gradual recovery thereafter (Table 1).

**Table 1:** Serial complete blood count during the illness and recovery

Hospital Day	Total Leucocyte Count (reference range: 4000-12000 cells/microlitre)	Haemoglobin (reference range: 11-14g/dl)	Haematocrit (reference range: 37-47%)	Platelets (reference range: 150-450k)
Day 0 (admission)	1920	10.7	32	182k
Day 1	2270	10	30	120k
Day 3	1490	10.6	32	11k
Day 5	1190	9.6	29	99k
Day 6	3820	10.8	32	85k
Day 7	3990	11.3	33	75k
Day 8	4000	11.5	35	110k
Day 9	4200	11.3	34	150k

**Figure 1:**
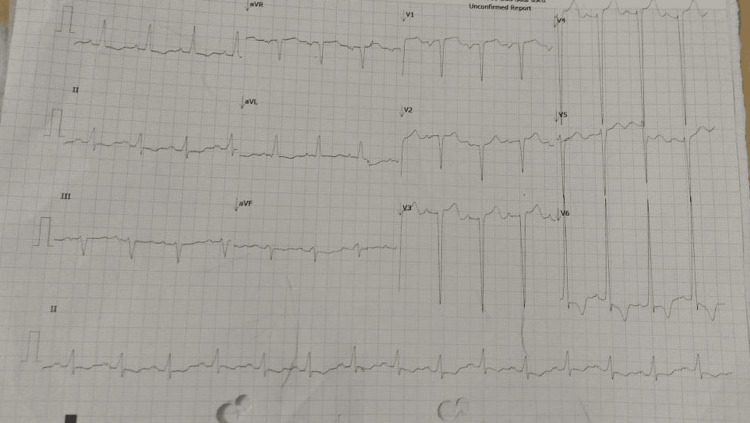
ECG showing left ventricular hypertrophy and ST-T changes

**Figure 2:**
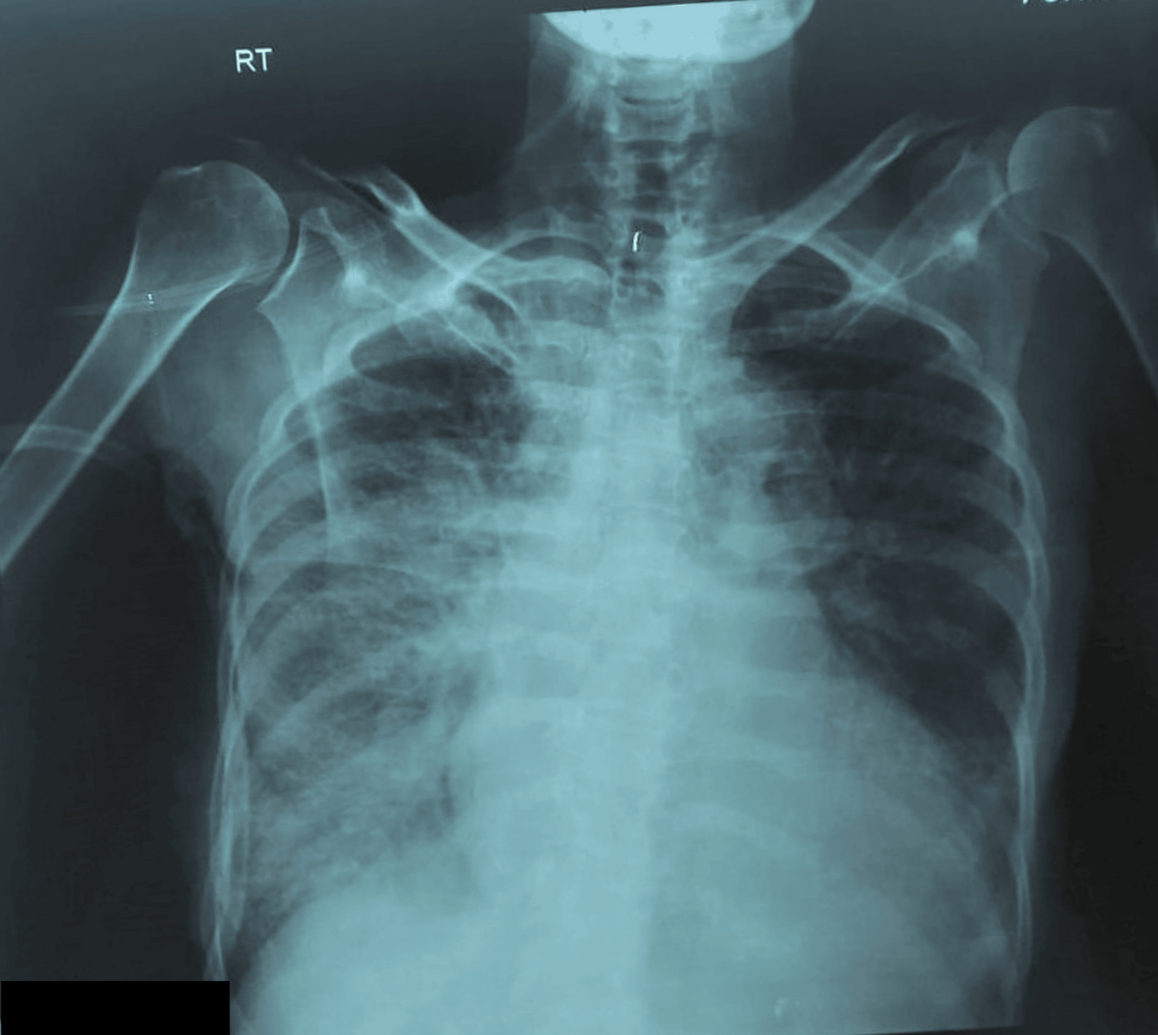
Chest X-ray showing pulmonary oedema and cardiomegaly

Cardiac biomarkers were elevated, with serial troponin I values rising from 0.31 ng/mL to 0.35 ng/mL and creatine kinase-myocardial band (CK-MB) increasing from 14.3 U/L to 17.4 U/L. ECG demonstrated nonspecific ST-T changes with left ventricular hypertrophy and strain pattern (Figure 1). Chest radiograph showed cardiomegaly with bilateral pleural effusions (Figure 2). Transthoracic echocardiography revealed a dilated left ventricle with severe left ventricular systolic dysfunction (ejection fraction (EF) 20%), moderate mitral regurgitation, mild pericardial effusion, and preserved right ventricular systolic function (Table 2). Abdominal ultrasound confirmed bilateral pleural effusion without evidence of hepatic or renal pathology. COVID-19 and influenza screens were negative, and the CT pulmonary angiogram did not show any pulmonary embolism.

**Table 2:** Echocardiogram readings showing reduced ejection fraction LVPW: left ventricular posterior wall; TR: tricuspid regurgitation; Aov MG/PG: aortic valve mean gradient/pressure gradient; MV: mitral valve; TAPSE: tricuspid annular plane systolic excursion; RVTDI: right ventricular tissue doppler imaging; IVRT: isovolumic relaxation time 1+: mild, 2+: moderate

Parameters	Patient Value	Reference range
Left ventricle end diastolic diameter	68	35-55 mm
Left ventricle end systolic diameter	59	25-41 mm
IVS Thickness end systolic	7.6	6-14 mm
Thickness end diastolic	7	6-11 mm
LVPW end systolic	6.5	6-14 mm
LVPW end diastolic	6	6-11 mm
Right Ventricle	31	7-25 mm
Right atrium	38	26-38 mm
Aortic Root Dimension	36	20-37 mm
Left Atrial Dimension	45	19-39 mm
Aortic Valve Opening	14	15-27 mm
Ejection Fraction	20	55-70%
Aortic Peak flow velocity	1.12	0.9-1.7
Peak TR gradient	29	-
Aov MG/PG Gradient	-	-
MV MG/PG Gradient	-	-
E/E'	-	-
TAPSE	16	-
RVTDI	0.9	-
E/A Ratio	> 1	-
IVRT	60 86 ms	-
Deceleration Time	167 - 231 ms	-
Valvular Regurgitation	-	-
Mitral Regurgitation	2+	
Tricuspid Regurgitation	1+	
Aortic Regurgitation	2+	
Pulmonic Regurgitation	-	

Differential diagnoses

In a patient presenting with acute febrile illness, thrombocytopenia, dyspnoea, and chest discomfort, the initial differential diagnoses included dengue fever with plasma leakage, viral myocarditis, community-acquired pneumonia, and sepsis-related cardiomyopathy. Given the respiratory findings and raised jugular venous pressure, acute heart failure secondary to viral myocarditis and dengue-associated capillary leak syndrome were considered most likely. Other viral etiologies of myocarditis, including COVID-19, influenza, and enterovirus, were also entertained but deemed less likely due to negative testing and the clinical scenario.

Management

The patient was managed as a case of dengue haemorrhagic fever with myocarditis. She was admitted to the intensive care unit (ICU) to closely follow her hemodynamic and fluid balance status. Intravenous fluids were given cautiously to avoid fluid overload (normal saline 200 mL every six hours, adjusted for clinical response and urine output). Supportive treatment included antipyretics, supplemental oxygen, and careful monitoring of vital signs, urine output, and haematocrit. The cardiology team was involved early in management. No inotropes were required, and diuretics were avoided in view of borderline intravascular volume status.

Outcome and follow-up

As her respiratory symptoms and oxygen saturation improved, she was transferred to the general medical ward for continued observation. Her pedal oedema resolved completely. Platelet counts and haematocrit normalized over the following week. After clinical stabilization and improvement in effort tolerance, she was discharged home with outpatient follow-up.

At the follow-up three weeks after discharge, she remained clinically stable without exertional dyspnoea or chest discomfort. Repeat transthoracic echocardiography showed significant improvement in left ventricular systolic function, with EF rising from 20% during the acute phase to 40%. No residual pericardial effusion was noted. She was advised to have ongoing follow-up with the cardiology team and counselling regarding fluid management and early presentation in case of recurrence of any symptoms of heart failure.

## Discussion

Dengue myocarditis is an uncommon but increasingly recognized manifestation within the expanded dengue syndrome. Its pathogenesis is multifactorial, involving both direct viral invasion of cardiomyocytes and immune-mediated inflammatory injury that can lead to transient or, at times, fulminant cardiac dysfunction. In a prospective cohort study by Salgado et al. of 102 children with dengue haemorrhagic fever, it was seen that the dengue virus was able to infect cardiomyocytes and cardiac endothelium, eliciting a robust inflammatory response [6]. The study showed marked intracellular calcium dysregulation in infected myocytes, supporting a direct viral cytopathic mechanism underlying dengue-associated myocarditis.

In our case, dengue myocarditis was diagnosed clinically based on compatible features: thrombocytopenia, positive dengue NS1 antigen, elevated cardiac biomarkers, and severe left ventricular systolic dysfunction (EF 20%) on echocardiography, after excluding alternative causes. Cardiac MRI and biopsy were unavailable, reflecting a resource-limited context; nevertheless, the combination of biochemical and echocardiographic evidence was used as per European Society of Cardiology guidelines for clinically suspected myocarditis (clinical presentation, raised cardiac biomarkers, and echocardiographic features of global LVH) [7]. With meticulous supportive care and cautious fluid resuscitation, the patient achieved complete symptomatic recovery, with an improvement in EF to 40% on follow-up.

Similar patterns of dengue-associated myocardial involvement have been documented across the literature. Sud et al. identified myocarditis using troponin and echocardiography, demonstrating that clinically significant cardiac injury can be recognized even without advanced imaging [8]. In other reports, Adams et al. [9] and Cristodulo et al. [10] confirmed dengue myocarditis with the help of cardiac MRI, illustrating its diagnostic precision in resource-rich settings. More severe forms of dengue with cardiac involvement, including refractory cardiogenic shock managed with extracorporeal membrane oxygenation, were reported by Teysseyre et al. [11] and fatal arrhythmias by Pramudyo et al. [12]. Compared with these reported cases, our patient’s reversible systolic dysfunction emphasizes the self-limiting nature of non-fulminant dengue myocarditis and the potential for full recovery when managed in a timely manner with early diagnosis.

Limitations

In the absence of cardiac MRI or endomyocardial biopsy, a definitive histopathological diagnosis of dengue myocarditis could not be established. Given these limitations, the diagnosis relied on clinical features, cardiac biomarkers, and echocardiographic findings. Long-term follow-up beyond the early recovery period was also limited. Despite these constraints, the case highlights that careful clinical evaluation and recognition, coupled with basic investigations, can guide effective management in low-resource settings.

Learning points

Dengue myocarditis should be considered when a patient with dengue infection develops unexplained dyspnoea or hypotension or has an investigation pointing towards cardiac enlargement. In the absence of advanced diagnostics such as cardiac MRI or biopsy, a combination of elevated cardiac biomarkers and echocardiographic evidence of global systolic dysfunction can provide a reasonable basis for a clinically suspected diagnosis of dengue-related cardiac involvement. Early recognition and careful supportive management, particularly judicious fluid resuscitation and close hemodynamic monitoring, are crucial and can result in full recovery even in resource-limited settings.

## Conclusions

This case reinforces that dengue myocarditis can occur even in the absence of classic dengue warning signs and may manifest primarily as cardiac failure. In resource-limited settings, early clinical suspicion becomes crucial, as inappropriate fluid administration-commonly used in dengue management-can exacerbate cardiac dysfunction rather than improve it. Early supportive care guided by simple and widely accessible investigation tools, such as troponin testing and bedside echocardiography, can help clinicians recognize cardiac involvement early in the disease process and adjust their management accordingly. Increasing awareness of dengue-related cardiac involvement and the use of practical diagnostic approaches are crucial for reducing preventable morbidity and mortality in endemic, resource-constrained regions.

## References

[1] Weng SL, Hung FY, Li ST (2025). Dengue epidemiology in 7 Southeast Asian countries: 24-year retrospective multicountry ecological study. Interact J Med Res.

[2] (2009). World Health Organization. Dengue: Guidelines for Diagnosis, Treatment, Prevention and Control. World Health Organization. Dengue: Guidelines for Diagnosis, Treatment, Prevention and Control.

[3] Gulati S, Maheshwari A (2007). Atypical manifestations of dengue. Trop Med Int Health.

[4] Wali JP, Biswas A, Chandra S (1998). Cardiac involvement in dengue haemorrhagic fever. Int J Cardiol.

[5] Simmons CP, Farrar JJ, Nguyen vV, Wills B (2012). Dengue. N Engl J Med.

[6] Salgado DM, Eltit JM, Mansfield K (2010). Heart and skeletal muscle are targets of dengue virus infection. Pediatr Infect Dis J.

[7] Schulz-Menger J, Collini V, Gröschel J (2025). 2025 ESC guidelines for the management of myocarditis and pericarditis. Eur Heart J.

[8] Sud R, Agarwal N, Aishwarya V (2023). A case series of dengue myocarditis: a complication observed in dengue patients. Cureus.

[9] Adams CD, Syro D, Llano JF, Betancur JF (2021). Myocarditis: an uncommon manifestation of dengue fever infection. BMJ Case Rep.

[10] Cristodulo R, Luoma-Overstreet G, Leite F (2023). Dengue myocarditis: a case report and major review. Glob Heart.

[11] Teysseyre L, Levy Y, Renou A (2020). Case report: refractory shock due to fulminant dengue myocarditis treated with venoarterial extracorporeal membrane oxygenation: a report of four cases. Am J Trop Med Hyg.

[12] Pramudyo M, Putra IC, Iqbal M (2024). Clinically suspected acute right ventricular fulminant dengue myocarditis masquerading with dual lethal arrhythmias: a case report. J Med Case Rep.

